# Uniform cobalt nanoparticles embedded in hexagonal mesoporous nanoplates as a magnetically separable, recyclable adsorbent

**DOI:** 10.3762/bjnano.9.168

**Published:** 2018-06-13

**Authors:** Can Zhao, Yuexiao Song, Tianyu Xiang, Wenxiu Qu, Shuo Lou, Xiaohong Yin, Feng Xin

**Affiliations:** 1 School of Chemical Engineering and Technology, Tianjin University, Tianjin 300350, China; 2 Tianjin Key Laboratory of Organic Solar Cells and Photochemical Conversion, School of Chemistry and Chemical Engineering, Tianjin University of Technology, Tianjin 300384, China

**Keywords:** absorbent regeneration, CoAl layered double hydroxide, efficient adsorbent, hexagonal nanoplates, magnetic nanoparticles, polydopamine

## Abstract

Novel hexagonal nanoplates (NPLs) comprised of mesoporous carbon containing imbedded magnetic Co nanoparticles (CoAl_2_O_4_ phase) are prepared through direct carbonization of polydopamine (PDA)-coated CoAl layered double hydroxide (LDH). A uniform PDA coating initially covers the surface of LDH by dopamine self-polymerization under mild conditions. Well-dispersed Co nanoparticles are formed in the NPLs by the partial reduction of cobalt from Co^2+^ to Co^0^ with surface carbon during the heat treatment process. The surface morphology and specific surface area of the as-prepared NPLs can be tailored by adjusting the initial dopamine concentration and carbonization temperature. The mesoporous NPLs exhibit excellent sorption of rhodamine B (RhB) dye and fast magnetic separation in aqueous solution. Over 95% of RhB can be adsorbed within 2 min and the adsorption reaches equilibrium after about 30 min. The maximum adsorption capacity approaches 172.41 mg/g. After regeneration, this adsorbent can be recycled easily by magnetic separation and still possess good adsorption capacity for RhB removal, even after five cycles.

## Introduction

Nanocomposites with unique electronic, mechanical, magnetic, and physicochemical properties have gained much attention because of their large specific surface area and high surface energy [1–2]. In the past few decades, researchers showed great interest in designing various nanocomposites for many potential applications, among which water pollution treatment has become an important issue for human health and environmental protection [3–5]. A large number of researches have demonstrated that carbon-based nanocomposites can serve as effective adsorbents for wastewater puriﬁcation [6–9]. The introduction of transition metal nanoparticles into a mesoporous carbon matrix to form transition-metal-loaded mesoporous carbon nanocomposites (TM-MCNs) has proved to be one of the most effective strategies for enhancing their adsorption performance in aqueous solutions [10–12]. Torad et al. [13] reported the high adsorption capacity of porous carbon particles with magnetic Co nanoparticles towards methylene blue (MB) dye. They attributed this to the formation of stable bi-dentate complexes between the Co particles and MB dye. The Co nanoparticles in the carbon-based nanocomposites not only enhance the adsorption ability, but also help to separate the adsorbent owing to their strong magnetic response.

Generally, Co nanoparticles can be introduced into mesoporous carbon matrices through a simple impregnation process or direct mixing of the carbon precursor with metal moieties [14–15], where the loaded Co nanoparticles are usually randomly dispersed in the mesoporous walls or aggregated with each other to form large particles. Although metal-organic frameworks (MOFs) with tunable cavities and tailorable chemistry have been demonstrated as precursors to generate TM-MCNs with various morphologies [16–17], it is still a significant challenge to prepare two-dimensional (2D) TM-MCNs derived from MOFs.

TM-MCNs with 2D morphology are of critical significance due to their unprecedented physicochemical properties among mesoporous carbon-based nanocomposites with multiple and distinct morphologies. Layered double hydroxides (LDHs) have attracted much attention recently because of their low cost, unique 2D structure, and tunable chemical composition [18–19]. They are a typical class of inorganic lamellar clays with dual-metal cations (e.g., CoAl) and anions or guest molecules within the interlayer region. The reduction of metal cations uniformly distributed in the lattice of LDHs by reducing agents to form well-dispersed metal nanoparticles in their framework has become a quite possible strategy for introduction of metal nanoparticles onto the support. The metal cations might migrate across the interlayer space during the thermal reduction process at high temperature due to the weak van der Waals forces between the interlayer of the LDH. Thus, LDHs possess great potential, serving as ideal precursors for the preparation of 2D TM-MCNs.

To achieve the reduction of metal cations by carbon in LDH lattices, a suitable carbon precursor is required. Dopamine (DA), a small biomolecule containing both amino and catechol functional groups, can be used as a type of carbon precursor with a high carbonization yield even at a high carbonization temperature [20]. More notably, it can self-polymerize under weak basic conditions at room temperature [21] and form a uniform coating [22] on almost any surface. The obtained polydopamine (PDA)-coated composite materials are expected to show excellent performance in the preparation of TM-MCNs.

**Scheme 1:**
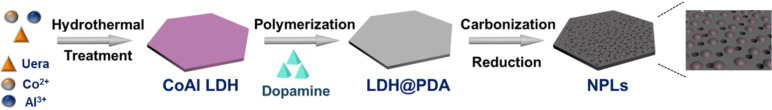
Schematic illustration of the formation process of the hexagonal magnetic mesoporous nanoplates.

Here we report a facile approach for the preparation of novel hexagonal nanoplates (NPLs) containing magnetic Co nanoparticles (in CoAl_2_O_4_ phase) and porous carbon by carbonizing PDA-coated CoAl LDH, which can be used as an adsorbent for efficient removal of RhB dye from aqueous solutions. As illustrated in Scheme 1, firstly, CoAl LDH is synthesized with urea through a hydrothermal method. Subsequently, the LDH@PDA composite is obtained by coating CoAl LDH with PDA through self-polymerization of dopamine in tris buffer solution at room temperature. Finally, the carbonization of LDH@PDA leads to the formation of the hexagonal NPLs containing magnetic Co (CoAl_2_O_4_) and mesoporous carbon originating from the CoAl LDH shape. The effects of different DA concentrations and carbonization temperatures on the NPLs are systematically investigated. This process has the following characteristics: (i) Co nanoparticles are evenly distributed in the NPLs by the reduction of uniformly distributed Co^2+^ ions in the lattice of CoAl LDH during the carbonization process; (ii) the NPLs maintain the original LDH hexagonal shape and possess relatively high porosity, which is produced by the consumption of surface carbon during the thermal reduction of Co^2+^ ions with carbon from the carbonization of dopamine; (iii) hexagonal mesoporous NPLs show a strong magnetic response due to the well-dispersed Co nanoparticles embedded in the carbon layer; and (iv) the obtained hexagonal magnetic mesoporous NPL displays a larger adsorption capacity towards removal of RhB from water than observed in other adsorbents [23–30].

## Experimental

### Materials and chemicals

Cobalt chloride hexahydrate, aluminum chloride hexahydrate, urea, 3-hydroxytyramine hydrochloride, and 2-amino-2-hydroxymethylpropane-1,3-diol (tris) were purchased from Aladdin Ltd. (Shanghai, China). Ethanol, hydrochloric acid, and rhodamine B were obtained from Yuanli Reagent Co., Ltd. (Tianjin, China). All chemicals were of analytical grade purity and used without any further puriﬁcation. Ultrapure water with a resistivity of 18.25 MΩ·cm was used in all experiments.

### Synthesis of CoAl layered double hydroxide

CoAl LDH was synthesized via a simple hydrothermal method using urea as the alkali source. In a typical process, CoCl_2_·6H_2_O, AlCl_3_·6H_2_O, and urea were dissolved in 100 mL ultrapure water with the concentration of 10 mM, 5 mM, and 35 mM, respectively. Then, the mixture was hydrothermally treated at 100 °C for 24 h in a 100 mL teﬂon-lined autoclave. After naturally cooling to room temperature, the pink solid product was collected by filtration, washed with ultrapure water three times, ethanol two times and then dried at room temperature.

### Preparation of the hexagonal magnetic mesoporous nanoplates

The obtained CoAl LDH (0.10 g) and dopamine hydrochloride (0.10, 0.15, 0.20, 0.25, and 0.30 g) were added into 100 mL tris buffer (10 mM) solution. The pH of the above mixture was adjusted by 1 M HCl to 8.5, which resulted in the polymerization of dopamine. After vigorously stirring for 12 h, the dark precipitate (LDH@PDA) was carefully collected by centrifugation and washed with deionized water several times to remove unreacted dopamine monomers, followed by drying in vacuum overnight. Based on the concentration of dopamine hydrochloride added in the coating process, the samples were denoted as LDH@PDA-*n*, where *n* stands for the concentration of dopamine hydrochloride. For example, LDH@PDA-2.5 means the concentration of dopamine hydrochloride was 2.5 g/L. The obtained LDH@PDA-*n* samples were further placed in a tube furnace and thermally treated under N_2_ at 500, 650, and 800 °C for 2 h with a heating rate of 2 °C/min. The final products were labeled as NPLs-*n*-*x*, where *x* stands for the carbonization temperature. The thermal treatment of pure CoAl LDH was performed under N_2_ at 800 °C for 2 h with a heating rate of 2 °C/min and the obtained product was a layered double oxide (LDO). The carbonized NPLs were soaked in 1 M HCl solution for 24 h to dissolve the Co nanoparticles, followed by washing with ultrapure water and then drying at room temperature. The resultant solid was labeled NPLs-S.

### Characterization

Powder X-ray diffraction (XRD) patterns were collected on a Bruker/D8-Advance instrument with Cu Kα radiation (λ = 1.5406 Å) at a scanning rate of 4°/min from 10° to 70°. The morphology and structure of the as-prepared samples were observed by scanning electron microscopy (SEM, ZEISS, Germany) and transmission electron microscopy (TEM, JEM-2100F, Japan). Energy dispersive X-ray (EDX) analysis was recorded via an EDX spectrometer attached to the SEM. The functional groups on the surface of the samples were measured by FTIR spectra on a Nicolet Nexus FTIR spectrometer using the KBr method within the wavelength range of 400–4000 cm^−1^. Nitrogen adsorption–desorption isotherms were obtained at 77 K on an Autosorb–iQ2–MP nitrogen adsorption apparatus. The Brunauer–Emmett–Teller (BET) specific surface area of the samples was calculated at a relative pressure range of 0.05–0.2. The pore size distribution was determined using nonlocal density functional theory (NLDFT) with the slit pore model using the adsorption branch of the nitrogen isotherm. The magnetic properties of the NPLs were investigated by a vibrating sample magnetometer (VSM) with an applied magnetic ﬁeld between −20 kOe and 20 kOe at room temperature (SQUID-VSM, USA). Atomic force microscopy (AFM) was performed on an AFM instrument (NTEGRA Spectra, Russia) using tapping mode. The samples were deposited onto clean Si substrates and dried at 60 °C. UV–vis adsorption spectra were recorded on a UV-2550 (Shimadzu, Japan) instrument. X-ray photoelectron spectroscopy (XPS) was conducted on a PHA-5400 (SPECS, America) spectrometer. Raman spectra were performed on an In-Via Raman spectroscopy system (Renishaw, England) with excitation laser wavelengths of 532 nm.

### Adsorption experiments

The adsorption experiments were carried out as follows: 10 mg of the as-prepared NPL samples were dispersed in 50 mL solutions at various RhB concentrations from 10 to 100 mg/L in a beaker at natural pH (about 6.9). The mixtures were sealed and placed in a temperature controlled shaker with a constant shaking speed of 180 rpm at 25 °C. To investigate the adsorption behavior, 3 mL of sample was taken from the mixture after a certain period of time and the adsorbent was collected using an external magnet. The concentration of the remaining RhB in the solution was detected by the UV–vis spectrophotometer at 553 nm. The adsorption capacity (*Q*_e_) at equilibrium and the removal efﬁciency (*R*_e_, %) for RhB were calculated by Equation 1 and Equation 2, respectively.

[1]
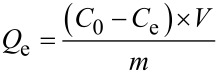


[2]
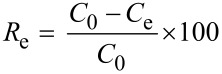


where *Q*_e_ (mg/g) is the amount of adsorbed dye per gram of adsorbent at equilibrium; *C*_0_ (mg/L) and *C*_e_ (mg/L) are the initial and equilibrium concentrations of RhB in the solution, respectively; *V* (L) represents the volume of RhB solution; *m* (g) is the weight of the adsorbent used.

## Results and Discussion

### Characterization of the hexagonal magnetic mesoporous nanoplates

**Figure 1:**
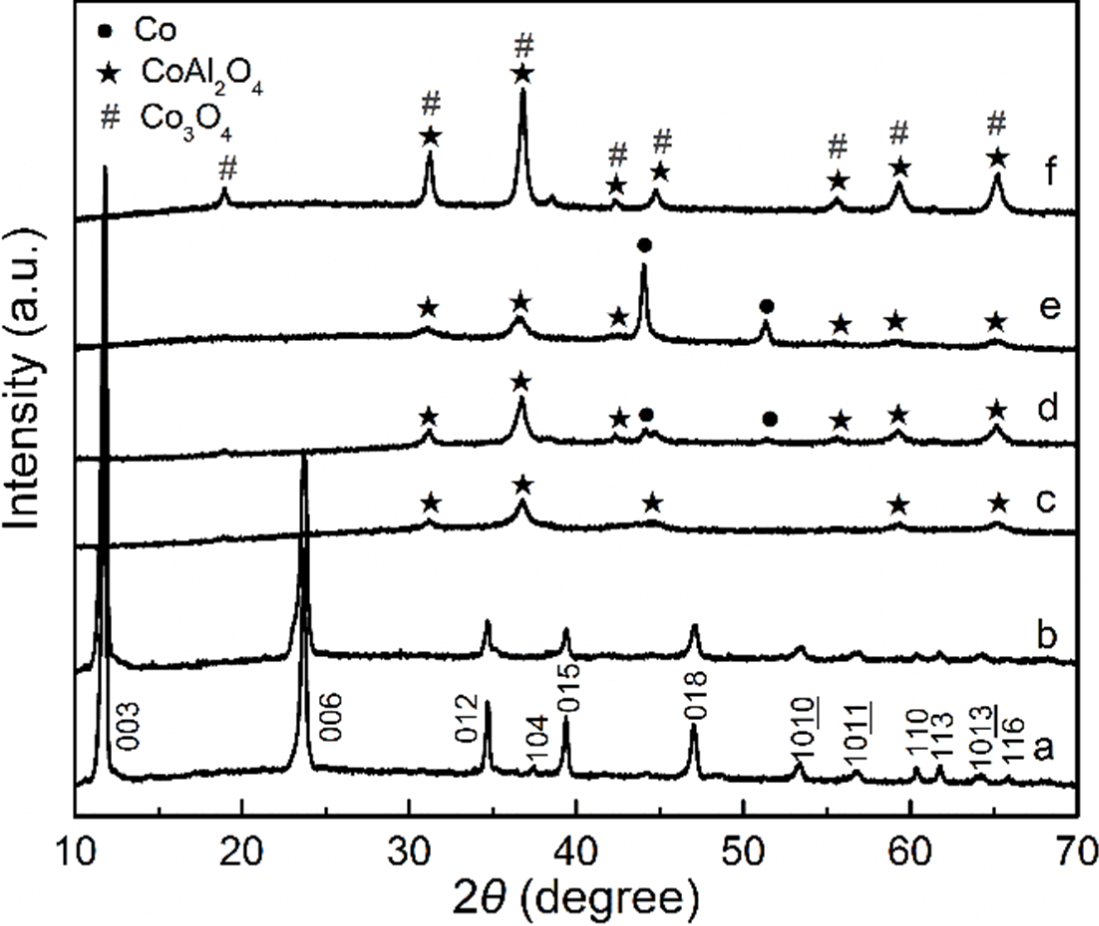
XRD patterns of: (a) pure CoAl LDH; (b) LDH@PDA-2.5 composite; (c–e) the NPLs prepared by carbonization of LDH@PDA-2.5 at 500, 650, and 800 °C for 2 h, respectively; (f) LDO (Co_3_O_4_ and CoAl_2_O_4_ mixture).

The crystallinity of the samples was confirmed by XRD and the results are given in Figure 1. The diffraction peaks in Figure 1a correspond well to the typical planes of CoAl LDH, suggesting its good crystallization and high purity. After coating with PDA, the resulting core–shell composite (LDH@PDA-2.5) displays characteristic diffraction peaks located at the same Bragg angles as pure CoAl LDH (Figure 1b), except the peak intensities become weaker, owing to the presence of the PDA coating layer. After the heat treatment of LDH@PDA-2.5 at 500 °C (Figure 1c), the sample shows diffraction peaks which belong to CoAl_2_O_4_. Two weak reﬂections at 44.2° and 51.4° begin to appear at 650 °C (Figure 1d). They become much stronger at 800 °C (Figure 1e) which are assigned to the (111) and (200) planes of face-centered cubic (fcc) metallic Co phase (JCPDS No.15-0806), respectively. This indicates that Co^2+^ ions in the lattice of LDH could be partially reduced to metallic Co by carbon during the carbonization of PDA above 650 °C. After calcination of pure CoAl LDH in a nitrogen atmosphere at 800 °C, only Co_3_O_4_ and CoAl_2_O_4_ mixtures are obtained (Figure 1f).

The successful coating of PDA on the surface of CoAl LDH is verified by FTIR spectra (Figure S1 in Supporting Information File 1). The characteristic adsorption bands at 1355 cm^−1^ and 790 cm^−1^ are assigned to the ν_3_ vibration and bending modes of CO_3_^2−^ within the interlayer of CoAl LDH, respectively (Figure S1a, Supporting Information File 1). The FTIR spectrum of LDH@PDA-2.5 shows two additional adsorption peaks at 1506 cm^−1^ and 1616 cm^−1^ (Figure S1b, Supporting Information File 1), which are consistent with the indole or indoline structures of PDA, indicating the successful coating of PDA on the surface of LDH [31–32].

**Figure 2:**
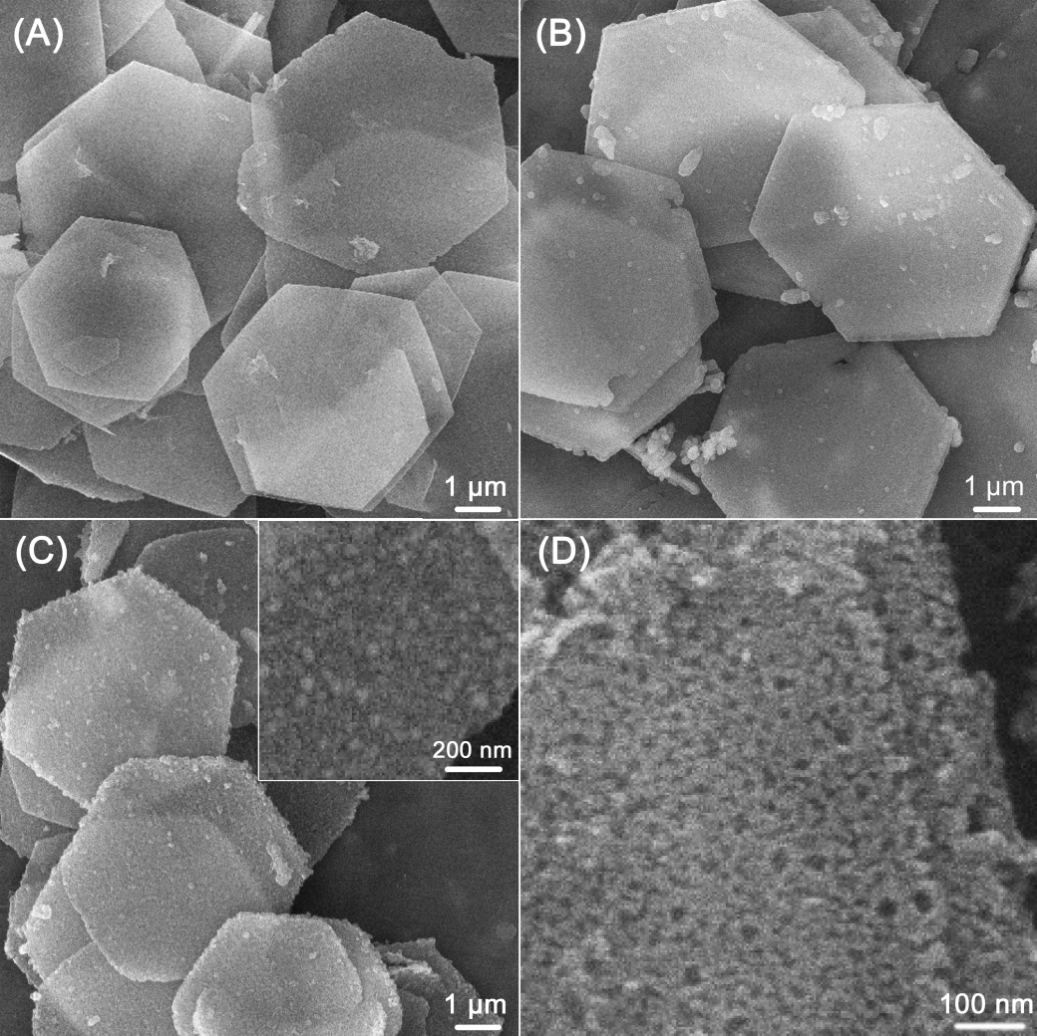
SEM images of: (A) pure CoAl LDH; (B) LDH@PDA-2.5 composite; (C) NPLs-2.5-800, the inset shows a high-magnification SEM image; (D) NPLs-S prepared by removal of Co nanoparticles embedded in NPLs-2.5-800 with HCl etching.

Figure 2A shows a SEM image of CoAl LDH which presents hexagonal platelets with a lateral size as large as 3–5 μm. It is confirmed that the PDA layer is uniformly coated on the surface of LDH (Figure 2B). After carbonization of the LDH@PDA-2.5 composite at 800 °C for 2 h (NPLs-2.5-800), well-dispersed Co nanoparticles are observed on the surface (Figure 2C), which are formed by the reduction of Co^2+^ ions with carbon. Mesoporous carbon nanoplates are observed after removing Co nanoparticles by HCl etching (Figure 2D). A series of hexagonal NPLs prepared with different DA concentrations and carbonization temperatures are shown in Figure S2, Supporting Information File 1. PDA layers become more noticeable with the increasing concentration of DA (Figure S2A–D, Supporting Information File 1). After the heat treatment at 800 °C, Co nanoparticles embedded in the rough and porous carbon layer are observed (Figure S2E–H, Supporting Information File 1). The surface roughness is observed to reduce with decreasing carbonization temperature (Figure S2H–J, Supporting Information File 1), suggesting the formation of a smaller number Co nanoparticles, which is consistent with the XRD results in Figure 1. The EDS spectrum and the elemental analysis of the NPLs-2.5-800 sample are shown in Figure S3 and Table S1, respectively, in Supporting Information File 1. The amount of C is 8.13 wt %, indicating a thin carbon layer on the surface of NPLs-2.5-800. The AFM image in Figure S4 (Supporting Information File 1) reveals that the NPLs-2.5-800 sample retains the hexagonal shape with a lateral size as large as 5 μm and thickness of approximately 100 nm, which is slightly larger than the thickness of pure CoAl LDH reported in the literature [33].

**Figure 3:**
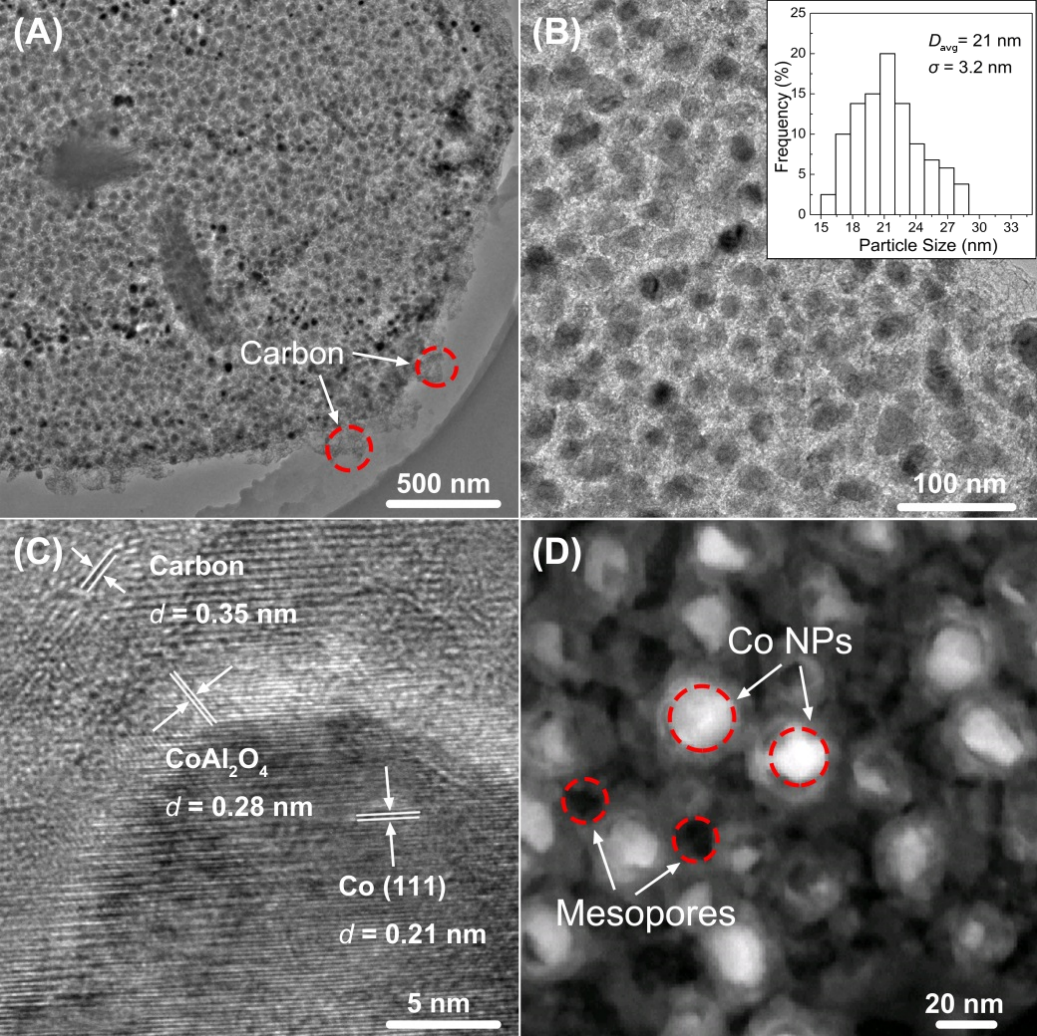
(A–C) TEM images of the hexagonal mesoporous sample NPLs-2.5-800, the inset in (B) shows the corresponding size histogram obtained by statistical analysis of over 80 Co nanoparticles. (D) HAADF-STEM image of the hexagonal mesoporous sample NPLs-2.5-800.

The morphology and structure of the as-prepared NPLs-2.5-800 sample are observed by TEM (Figure 3A–C) and STEM (Figure 3D). As shown in Figure 3A, well-dispersed Co nanoparticles on the surface of NPLs-2.5-800 are observed clearly. A thin carbon layer is found on the edge of the platelet. An HRTEM image shows Co nanoparticles with an average diameter of 21 nm that are embedded evenly in the carbon layer (Figure 3B). The measured *d*-spacing value of 0.21 nm in Figure 3C corresponding to the plane of a fcc Co crystal can be observed clearly, which is consistent with the XRD data in Figure 1. The Co nanoparticles are surrounded by a CoAl_2_O_4_ phase and a thin graphitized carbon layer, implying that the PDA@LDH composite is converted completely to the hexagonal NPLs-2.5-800 containing magnetic Co nanoparticles (CoAl_2_O_4_ phase) and porous carbon layer by partial reduction Co^2+^ ions in the lattice of LDH with carbon during carbonization process at 800 °C [15,34]. The Z-contrast image in HAADF-STEM image correlates strongly with the atomic mass. Metallic cobalt is a heavier element and thus appears as the bright contrast, whereas the mesopores generated by the consumption of surface carbon are observed as darker contrasting areas in Figure 3D. From the HAADF-STEM image in Figure S5A of Supporting Information File 1, and the corresponding elemental line profiles in Figure S5B, the C counts change very little, which indicates that the NPLs are covered by thin carbon layers. By contrast, the Co content reaches its maximum in the middle position (Figure S5B, Supporting Information File 1), corresponding to the bright contrast areas (Co nanoparticle) along the red line in Figure S5A. The result indicates that the Co nanoparticles are surrounded by C and CoAl_2_O_4_ phases. Elemental mapping images of the NPLs-2.5-800 sample indicate that the C, O, Co, and Al are uniformly distributed throughout the entire nanoplate framework (Figure S5C, Supporting Information File 1).

**Figure 4:**
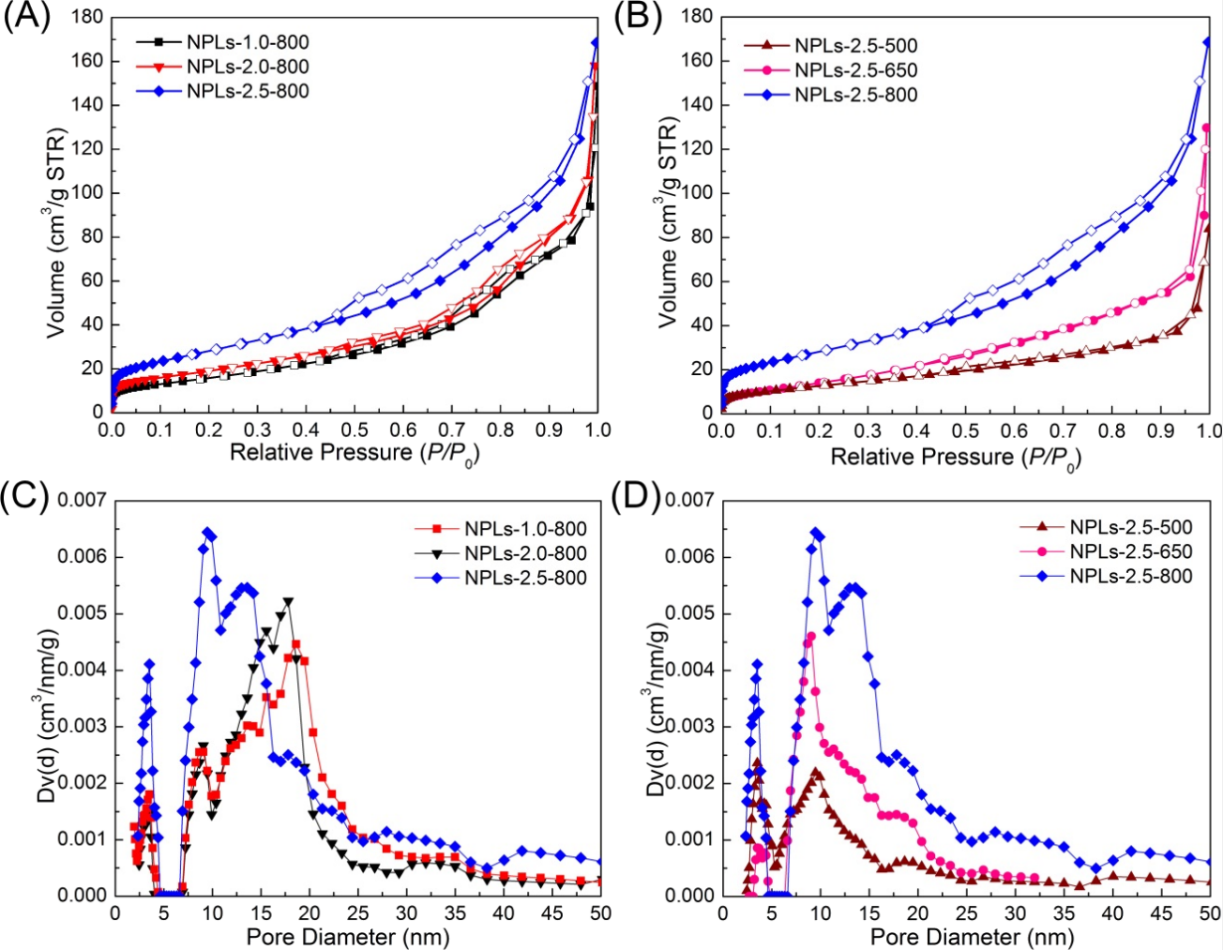
(A) and (B) Nitrogen adsorption–desorption isotherms of the NPLs prepared under different conditions. (C) and (D) Corresponding pore size distributions derived from the adsorption branches using the NLDFT method.

The nitrogen adsorption–desorption isotherms are measured to confirm the porosity of the hexagonal NPLs. As shown in Figure 4A, all isotherms of the samples exhibit a capillary condensation step at relative pressure of *p*/*p*_0_ = 0.45–0.9, known as a type IV(a) isotherm according to IUPAC classification [35]. The average pore size of all samples calculated from the adsorption branch using the NLDFT method is less than 25 nm, as shown in Figure 4C, which is in good agreement with the STEM observation (Figure 3D). After heat treatment at 800 °C, the BET surface area for the samples prepared at the initial dopamine hydrochloride concentrations of 1.0, 2.0, and 2.5 g/L is calculated to be 59, 70, and 124 m^2^/g, respectively (Table S2, Supporting Information File 1). When the initial concentration of dopamine hydrochloride is decreased, the average pore size increases from 9.5 nm to 18.6 nm (Figure 4C and Table S2 (Supporting Information File 1)) owing to the reduced amount of carbon source. An increase in the amount of carbon precursor would lead to more micro- and mesopores on the NPLs. The higher mesoporosity of the NPLs-2.5-800 sample could enhance accessibility of RhB, improving the adsorption performance. As shown in Figure 4D, the pore size distribution regions of three samples prepared using different carbonization temperatures are mainly located between ≈7–25 nm. The higher carbonization temperature would lead to larger mesopores owing to the consumption of the surface carbon layer for the Co^2+^ reduction above 650 °C.

**Figure 5:**
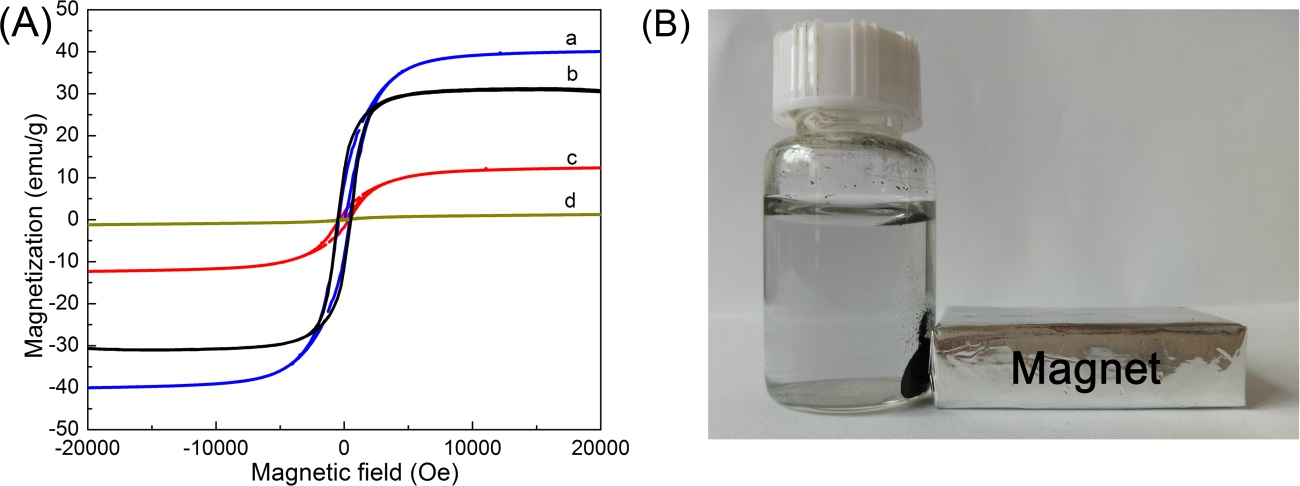
(A) Magnetization curves of the nanoplate samples prepared by carbonization of LDH@PDA at (a–c) 800 and °C (d) 650 °C for 2 h. LDH@PDA composites were prepared with different initial dopamine hydrochloride concentrations of: (a) 2.0, (b) 2.5, (c) 1.0 and (d) 2.5 g/L. (B) A photograph illustrating the physical separation of the adsorbent material (NPLs-2.5-800) from water in the presence of an external magnetic field (permanent magnet).

The magnetic properties of the NPL samples were investigated using a VSM with an applied magnetic ﬁeld of 20 kOe at room temperature. As shown in Figure 5A and Table S2 (Supporting Information File 1), the highest saturation magnetization value (40.1 emu/g) is found when the initial concentration of dopamine hydrochloride is 2.0 g/L after heat treatment at 800 °C (NPLs-2.0-800). Decreasing the carbonization temperature, the number of generated Co nanoparticles decreases, which can be observed in Figure 1c–e and Figure S2H–J (Supporting Information File 1), finally resulting in a low saturation magnetization value for NPLs-2.5-650. Additionally, more carbon covering the surface of NPLs will weaken the magnetic response of Co nanoparticles, leading to a lower saturation magnetization value (NPLs-2.5-800 vs NPLs-2.0-800) [36]. The large magnetic hysteresis loops for samples NPLs-2.0-800 and NPLs-2.5-800 in Figure 5A indicate their strong magnetic response to the varying magnetic field, which is highly favorable for fast separation after the adsorption process. Figure 5B shows that the black adsorbent material (NPLs-2.5-800) can be attracted by a magnet within 20 s, indicating the excellent of magnetic separation ability.

**Figure 6:**
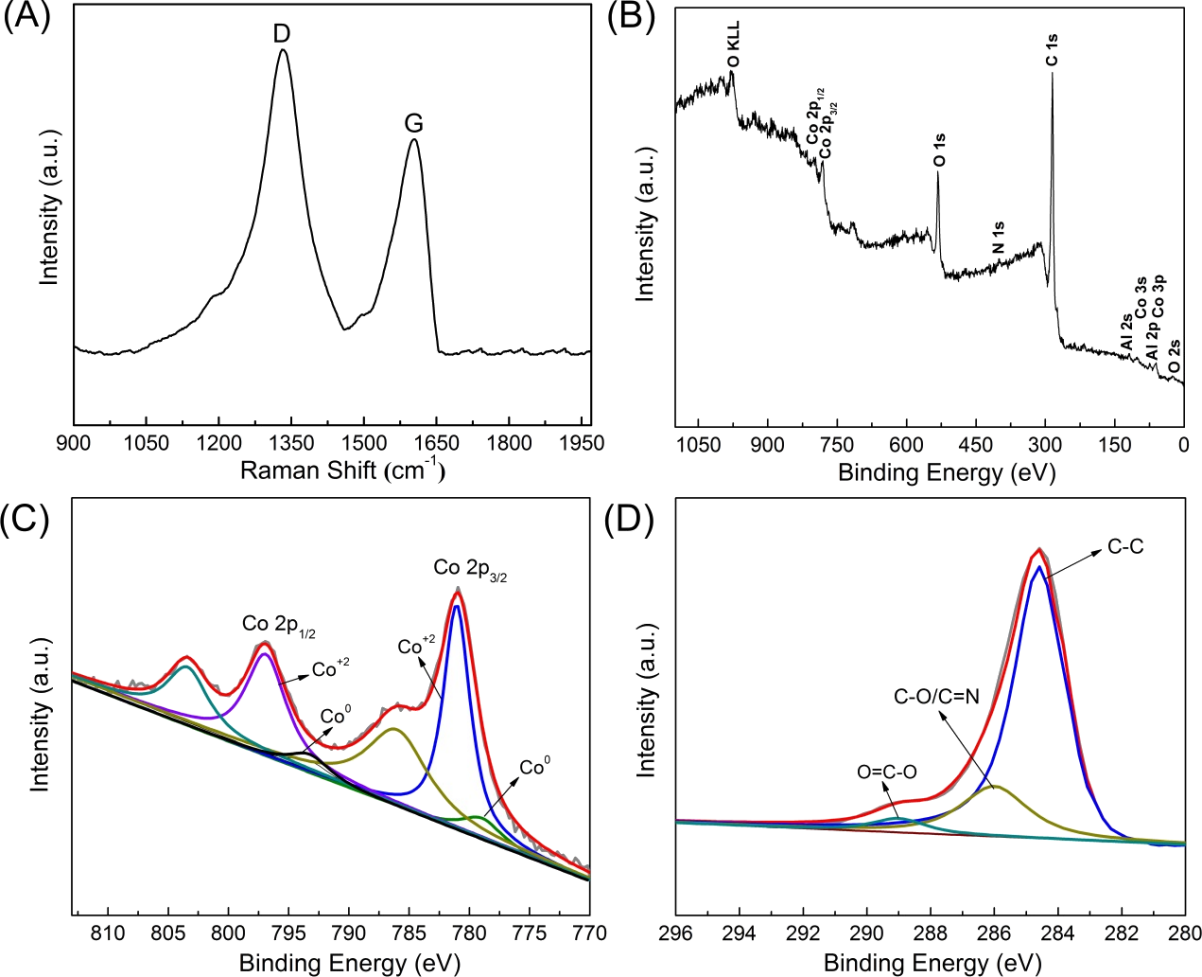
(A) Raman spectrum and (B–D) XPS spectra of the hexagonal magnetic mesoporous sample NPLs-2.5-800. (B) Full survey XPS spectrum, (C) high-resolution Co 2p spectrum, and (D) high-resolution C 1s spectrum.

The properties of carbon generated by carbonization of the surface PDA layer are illustrated in the Raman spectrum. As shown in Figure 6A, the typical D and G bands at around 1355 cm^−1^ and 1598 cm^−1^ are observed clearly, corresponding to the in-plane vibration mode of the sp^2^ carbon atoms in amorphous and graphitic carbon, respectively [22,37]. The intensity ratio of *I*_D_/*I*_G_ (*I* represents the intensity of the D and G peaks) for the hexagonal magnetic mesoporous sample NPLs-2.5-800 is determined to be 1.02, indicating most of the carbon is amorphous in structure [38]. XPS was used to investigate the surface composition of the sample NPLs-2.5-800 and the valence state of the elements. The elements Co, Al, C, O, and N can be clearly observed in the full survey XPS spectrum (Figure 6B). The two characteristic peaks located at 780.8 eV and 796.5 eV in the Co 2p spectrum are assigned to the 2p_3/2_ and 2p_1/2_ spin–orbit split peaks of Co (II) species, respectively (Figure 6C). Two small peaks at 786.4 eV and 803.5 eV are two satellite peaks of Co (II) species [39]. XPS is sensitive to atoms in the near-surface layer, hence only two weak peaks in Figure 6C ascribed to Co (0) are observed because most of the Co nanoparticles are surrounded by the spinel oxide and carbon matrix. The C 1s spectrum shows three peaks (Figure 6D) correlated with several types of C species (C–C at 284.6 eV, C=N/C–O at 286.6 eV, and O–C=O at 289.0 eV) [40], indicating that nitrogen-doped carbon is obtained.

### Adsorption properties of the hexagonal magnetic mesoporous nanoplates

**Figure 7:**
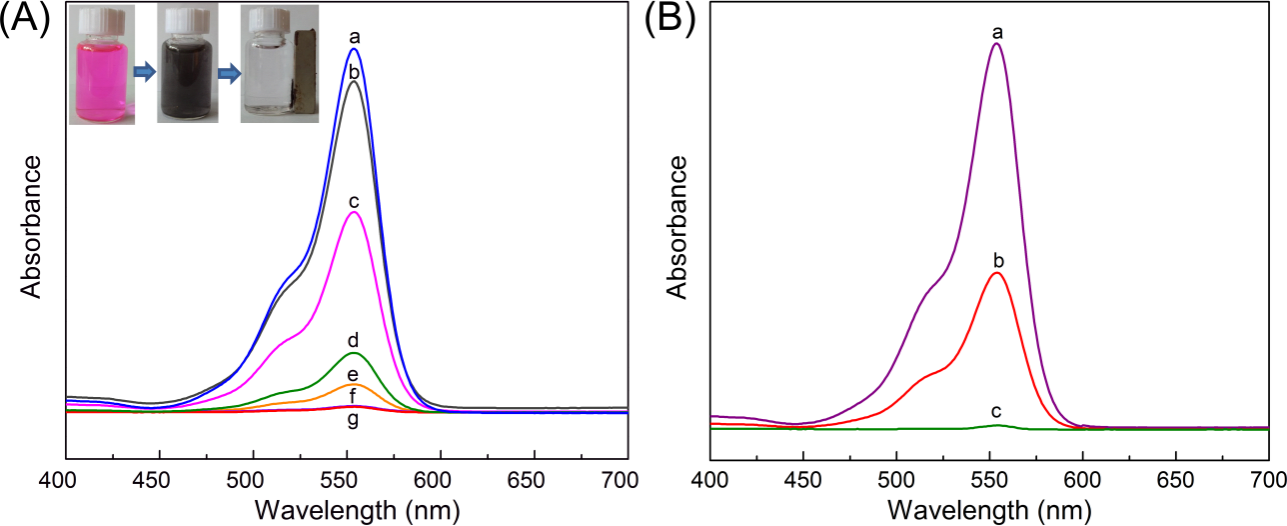
(A) UV–vis spectra of the solution after adsorption of RhB for 2 h in the absence of any adsorbents (a) and in the presence of the hexagonal NPLs prepared by carbonization of LDH@PDA at 800 °C for 2 h (b–g). LDH@PDA samples were prepared with different initial dopamine hydrochloride concentrations of: (b) 0, (c) 1.0, (d) 1.5, (e) 2.0, (f) 2.5, and (g) 3.0 g/L. (B) UV–vis spectra of the solution after adsorption of RhB for 2 h using the NPLs prepared by carbonization of LDH@PDA-2.5 at different temperatures for 2 h: (a) 500, (b) 650, and (c) 800 °C. The concentration of adsorbents is 0.2 g/L and the initial concentration of RhB solution is 25 mg/L.

Figure 7A shows the typical UV–vis adsorption of the NPLs prepared by varying the initial concentration of dopamine hydrochloride under otherwise identical conditions. The adsorption ability of the NPL samples gradually increases as the initial amount of dopamine hydrochloride increases. The highest adsorption capacity is found when high concentrations of dopamine hydrochloride are used (2.5 and 3.0 g/L). This result indicates that the increases in the exposed specific surface area and pore volume resulted from the increased amount of carbon precursor, which promotes the adsorption of RhB. In contrast, LDO obtained by calcination of pure CoAl LDH at 800 °C has the lowest adsorption capacity towards RhB due to the absence of a porous carbon layer and Co nanoparticles. Additionally, the carbonization temperature significantly affects the adsorption ability of the hexagonal NPLs. The adsorption ability reduces dramatically with the decrease in carbonization temperature (Figure 7B) at constant initial dopamine hydrochloride concentration (2.5 g/L). This might be due to the absence of Co nanoparticles and decreasing porosity at a relatively lower carbonization temperature (Figure 1c–e and Table S2, Supporting Information File 1).

**Figure 8:**
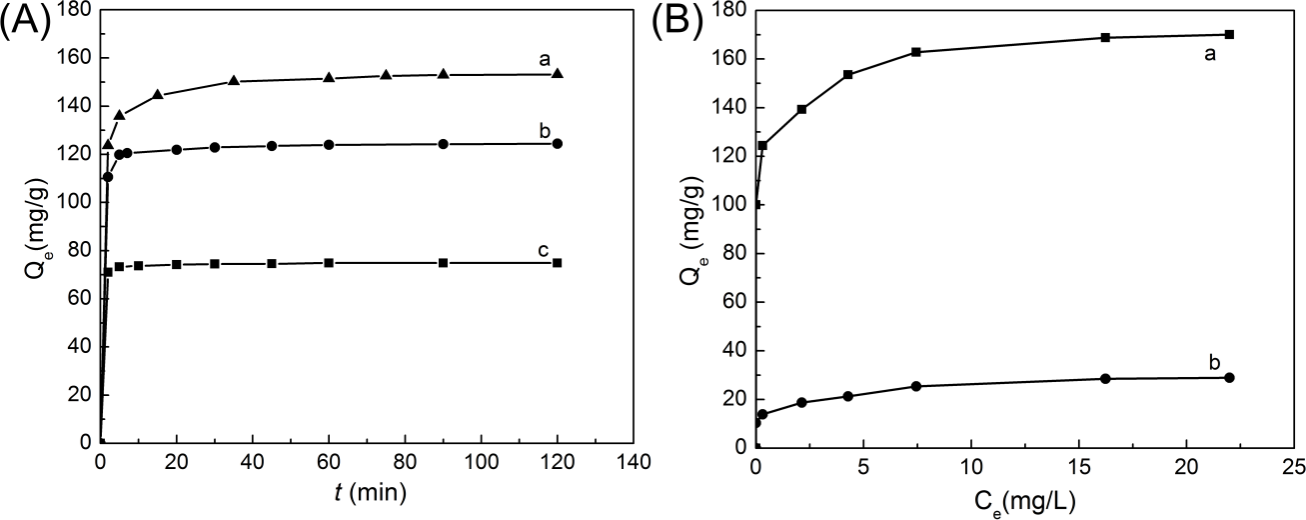
(A) Relationship between the adsorption ability of the hexagonal NPLs-2.5-800 sample and time at different initial RhB concentrations of: (a) 35, (b) 25, and (c) 15 mg/L. The concentration of adsorbents is 0.2 g/L. (B) Adsorption isotherms of the hexagonal NPLs-2.5-800 sample at 25 °C (a) before (with Co nanoparticles) and (b) after (Co nanoparticles removed) HCl etching.

The kinetics and isotherm models for the adsorption of RhB were investigated systematically. Figure 8A displays the adsorption capacity of the NPLs-2.5-800 sample at different initial RhB concentrations. It only takes less than 10 min to reach equilibrium at low initial RhB concentrations (15 and 25 mg/L), and it takes about 30 min to reach equilibrium at a high initial RhB concentration (35 mg/L). The adsorption isotherm of RhB on the NPLs-2.5-800 sample is well-fitted by the Langmuir isotherm model (Figure S6 and Table S3 in Supporting Information File 1). The calculated value of *Q*_max_ from the Langmuir isotherm model is 172.41 mg/g, which is very close to our experimental value of 170.05 mg/g. The NPLs-2.5-800 sample can quickly adsorb 95% of RhB dye from water within 2 min at a low initial RhB concentration (Figure 8A, parts (b) and (c)), indicating a quite fast adsorption rate (17.21 mg/g·min) is achieved. Adsorption behavior of RhB onto the NPLs-2.5-800 adsorbent is in good agreement with the pseudo-second-order kinetics model (Figure S7 and Table S4 in Supporting Information File 1). After removal of the Co nanoparticles in sample NPLs-2.5-800 with HCl, the NPLs-S sample exhibits poor adsorption capacity (28.82 mg/g) compared with the NPLs-2.5-800 sample (Figure 8B). This result indicates that the Co nanoparticles embedded in the NPLs play an important role in the adsorption of RhB dye, which can provide active sites for chemisorption of RhB via the formation of stable bi-dentate complexes [13,41]. Thus, the porous carbon layer on the surface of NPLs and the well-dispersed Co nanoparticles could both contribute to the adsorption performance. Table S5 in Supporting Information File 1 shows some recent research on the adsorption of RhB for various adsorbents. It is not difficult to see that the as-prepared NPLs-2.5-800 sample in our study displays relatively good adsorption performance for RhB dye.

**Figure 9:**
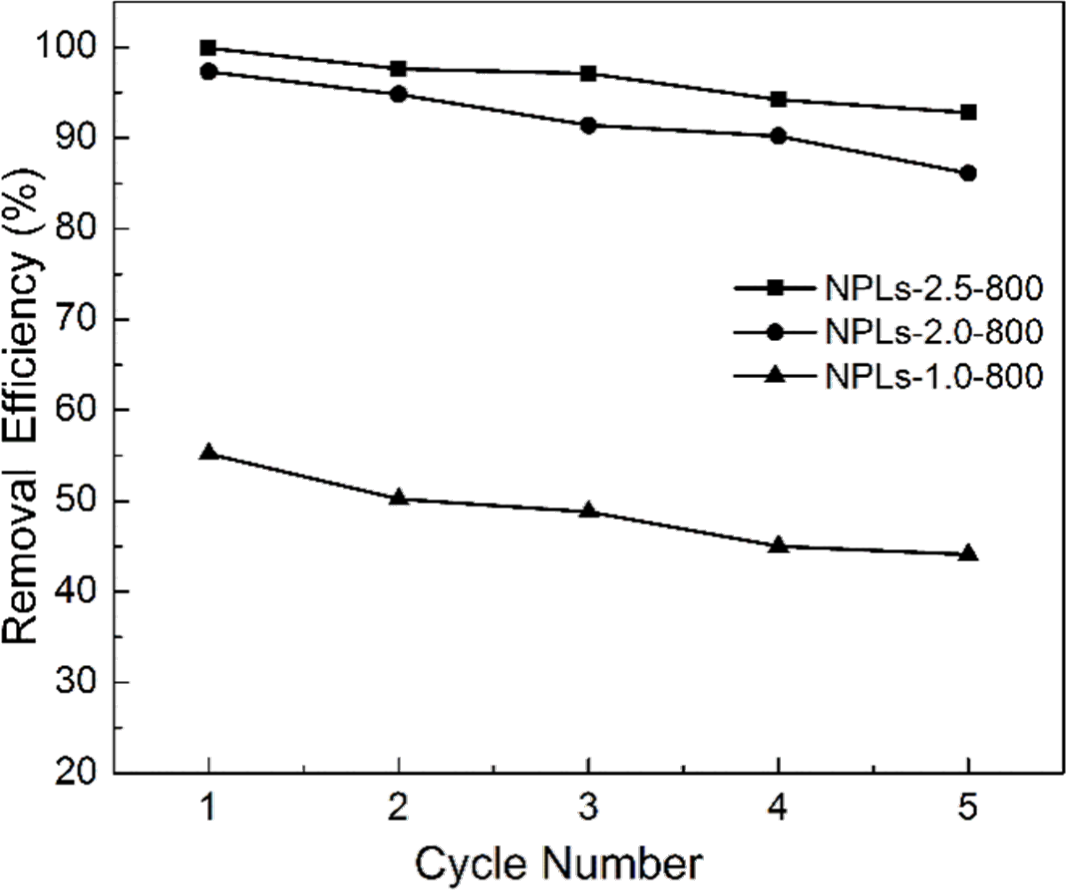
Recyclability of the NPLs adsorbents towards the removal of RhB. The concentration of adsorbents is 0.2 g/L, the initial concentration of RhB is 35 mg/L and the time of adsorption is 2 h.

The regeneration ability of the adsorbents is of great importance for practical application. In order to qualify as a good absorbent, the material must not only possess high adsorption efficiency but it should be easily recyclable to reduce the cost of the adsorption process. The desorption experiments are carried out by simply washing the adsorbents with ethanol three times. The volume of ethanol is 10 mL for 10 mg adsorbent each time. After the desorption process, the NPL samples are used for the removal of RhB from aqueous solutions for five cycles (Figure 9). It was found that the NPLs-2.5-800 adsorbent possesses the best adsorption capacity, where removal efficiency for RhB reaches 99.8% at first and decreases to 92.8% after five cycles. All samples have a small decrease of adsorption capacity after five cycles. The small loss of removal efficiency is likely due to the slightly reduced specific surface area and pore volume as shown in Figure S8 (Supporting Information File 1), which suggests that the unavoidable residual RhB molecules still exist in the NPLs-2.5-800 sample after the desorption process. Easy regeneration and good reusability make the hexagonal magnetic mesoporous NPLs potentially suitable for low-cost wastewater treatment.

## Conclusion

In summary, we have developed a novel approach to prepare hexagonal magnetic mesoporous nanoplates (NPLs) by direct carbonization of PDA-wrapped CoAl LDH. Owing to the even distribution of Co^2+^ in the LDH lattice and uniform PDA layer on the surface of LDH, the resultant NPLs possess a mesoporous structure and good distribution of Co nanoparticles formed by partially reducing the Co^2+^ with carbon after the heat treatment process. The pore characteristics and surface morphology of the hexagonal magnetic mesoporous NPLs can be tailored by adjusting the dopamine concentration and carbonization temperature. The NPLs also exhibit excellent adsorption ability for RhB dye due to the evenly distributed Co nanoparticles and mesoporous carbon layer. Additionally, this NPL adsorbent material can be quickly separated from aqueous solutions by using an external magnet due to their strong magnetic response. The merits of the as-prepared NPLs, including the good distribution of metal nanoparticles, strong magnetic response, fast adsorption rate, high adsorption capacity, and easy regeneration, provide opportunities for designing new composites with enhanced sorption behavior. This hexagonal magnetic mesoporous NPL composite is also expected to have significant potential applications in the other fields, such as energy storage, photo-electrocatalysis, and CO_2_ capture.

## Supporting Information

FTIR spectra of pure CoAl LDH and LDH@PDA-2.5 composite; SEM images of NPL samples prepared at different DA concentrations and carbonization temperatures; EDX spectrum, elemental analysis from EDX data, AFM image and HAADF-STEM images of the NPLs-2.5-800 sample; textural and magnetic properties of the as-prepared samples; parameters of adsorption isotherms and kinetics for removal of RhB by NPLs-2.5-800 sample; nitrogen adsorption–desorption isotherms and the corresponding pore size distributions for sample NPLs-2.5-800 before and after 5 cycles; comparison of adsorption capacity of various adsorbents for RhB dye removal.

File 1 Additional experimental data.
