# Supplementary material for: Uniform cobalt nanoparticles embedded in hexagonal mesoporous nanoplates as a magnetically separable, recyclable adsorbent
Source: Beilstein J Nanotechnol. 2018 Jun 13;9:1770–81. doi: 10.3762/bjnano.9.168 (PMC6009527; doi:10.3762/bjnano.9.168)
Supplement: File 1 — Additional experimental data. [file Beilstein_J_Nanotechnol-09-1770-s001.pdf]

Supporting Information

for

Uniform cobalt nanoparticles embedded in hexagonal mesoporous nanoplates as a magnetically separable, recyclable adsorbent

Can Zhao[1], Yuexiao Song[1], Tianyu Xiang[1], Wenxiu Qu[1], Shuo Lou[1], Xiaohong Yin[2] and Feng Xin[*1]

Address: [1]School of Chemical Engineering and Technology, Tianjin University, Tianjin 300350, China and [2]Tianjin Key Laboratory of Organic Solar Cells and Photochemical Conversion, School of Chemistry and Chemical Engineering, Tianjin University of Technology, Tianjin 300384, China

*Corresponding author

Email: Feng Xin - xinf@tju.edu.cn

Additional experimental data

Adsorption isotherms and kinetics of RB

Adsorption isotherms are significant data for investigating the adsorption mechanism and the interactions between contaminant and adsorbents. Our adsorption experiments for RB dye with different initial RB concentrations (10 to 100 mg/L) were proceed at initial pH (about 6.9), constant temperature (*T* = 25 °C) with a certain period of adsorption time (*t* = 2 h). The Langmuir isotherm model is selected in this study to analyze the equilibrium adsorption of RB onto the NPLs-2.5-800 adsorbent, which can be expressed as the following Eq. (S1)

$$\frac{C_e}{Q_e} = \frac{C_e}{Q_{max}} + \frac{1}{Q_{max}K_l} \qquad \text{(S1)}$$

where C_e is the equilibrium concentration of RB in the solution (mg/L); Q_e represents the equilibrium amount of adsorbed dye per unit mass of the NPLs-2.5-800 (mg/g); K_l (L/mg) and Q_{max} (mg/g) are the Langmuir adsorption constant associated with the energy of adsorption and maximum adsorption capacity, respectively.

Pseudo-first-order, pseudo-second-order, and Weber-Morris intraparticle diffusion models are used. They are denoted as the following Eq. (S2), (S3) and (S4), respectively.

$$ln(Q_e - Q_t) = ln\, Q_e - K_1 t \qquad \text{(S2)}$$

$$\frac{t}{Q_t} = \frac{1}{K_2 Q_e^2} + \frac{1}{Q_e} t \qquad \text{(S3)}$$

$$Q_t = K_3 t^{1/2} + C \qquad \text{(S4)}$$

where Q_e (mg/g) and Q_t (mg/g) are the amount of adsorbed RB at equilibrium and at contact time *t* (min), respectively. K_1 (min^{-1}) is the rate constant of pseudo-first-order and can be calculated from the plot of log(Q_e-Q_t) against *t* in Eq. (S2). K_2 is the rate constant of pseudo-second-order

(g/min·mg). K_3 (mg/g·min) is the intraparticle diffusion rate constant and C (mg/g) is another constant related to the boundary layer thickness.

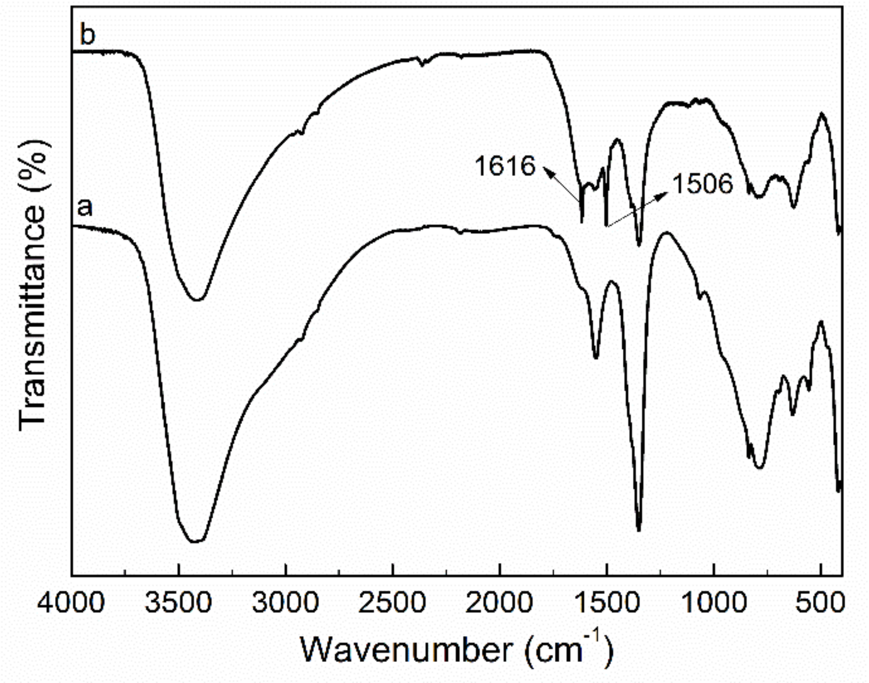

Figure S1: FTIR analysis of the samples: (a) pure CoAl LDH and (b) LDH@PDA-2.5 composite.

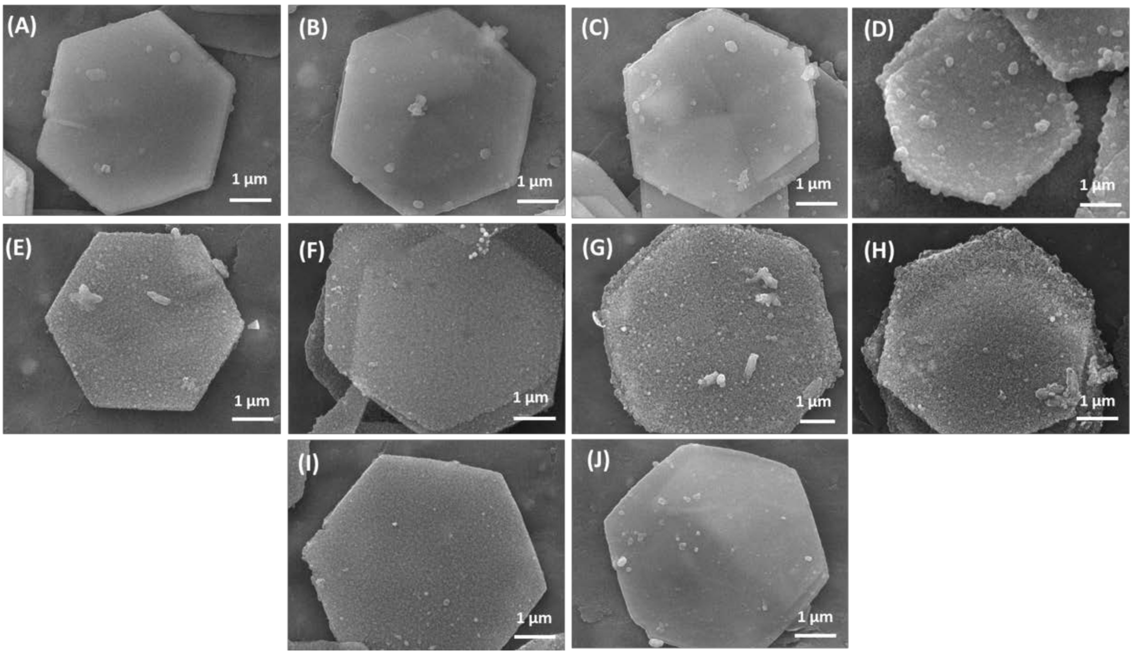

Figure S2: SEM images of samples: (A)–(D) LDH@PDA prepared with different initial dopamine hydrochloride concentrations: (A) 1.0, (B) 1.5, (C) 2.0, and (D) 3.0 g/L; (E)–(H) the NPLs prepared by carbonization of the above LDH@PDA at 800 °C for 2 h; (I)–(J) the NPLs prepared by carbonization of LDH@PDA-2.5 at different temperatures for 2 h: (I) 650 and (J) 500 °C.

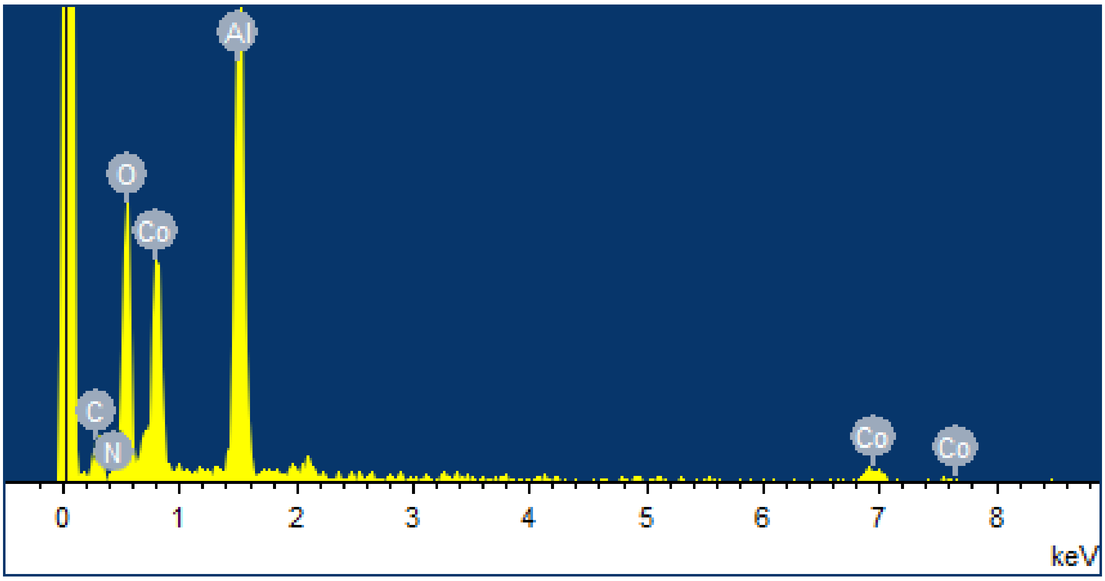

Figure S3: Energy-dispersive X-ray spectrum of the NPLs-2.5-800 sample.

Table S1: Elemental analysis of the NPLs-2.5-800 sample based on EDX data.

Elements	Weight (%)	Atom (%)
C	8.13	18.01
N	0.92	1.75
O	21.19	35.21
Al	25.48	25.08
Co	44.28	19.95

Table S2: Structural, textural, and magnetic properties of the as-prepared samples.

Samples	S_{BET} [a] (m^2/g)	S_{micro} [b] (m^2/g)	S_{meso} [c] (m^2/g)	V_p [d] (cm^3/g)	D [e] (nm)	Magnetic saturation value (emu/g)
NPLs-1.0-800	59	15	44	0.21	18.6	12.3
NPLs-2.0-800	70	21	49	0.24	17.8	40.1
NPLs-2.5-800	124	33	91	0.33	9.5	31.2
NPLs-2.5-650	56	10	46	0.23	9.1	1.3
NPLs-2.5-500	47	8	39	0.08	7.0	/

[a] S_{BET} (total surface area) is calculated using the BET method. [b] S_{micro} (micropore surface area) and [c] S_{meso} (mesopore surface area) are calculated calculated using the *t*-plot method. [d] V_p is the total pore volume of micro- and mesopores. [e] D is the mean pore size estimated by NLDFT method.

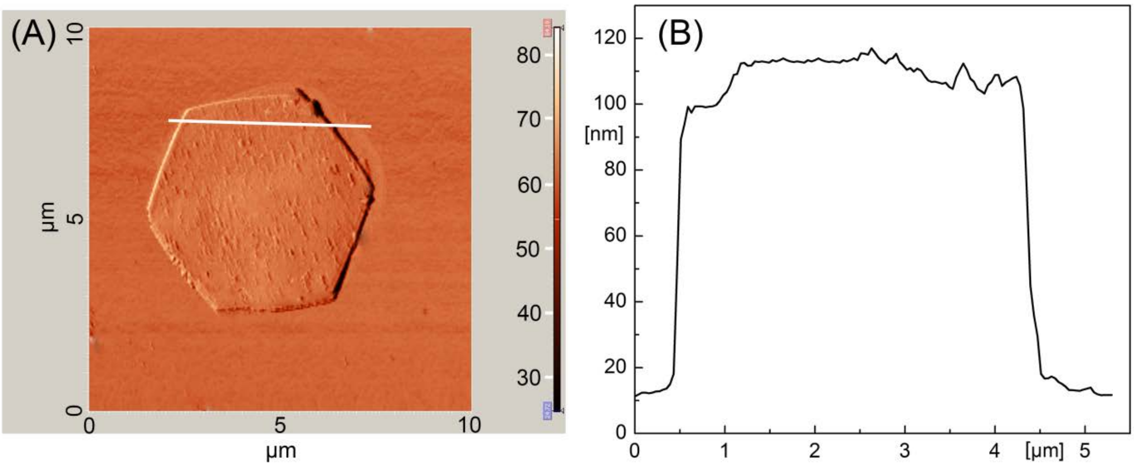

Figure S4: (A) AFM image and (B) height profile of the as prepared NPLs-2.5-800 sample.

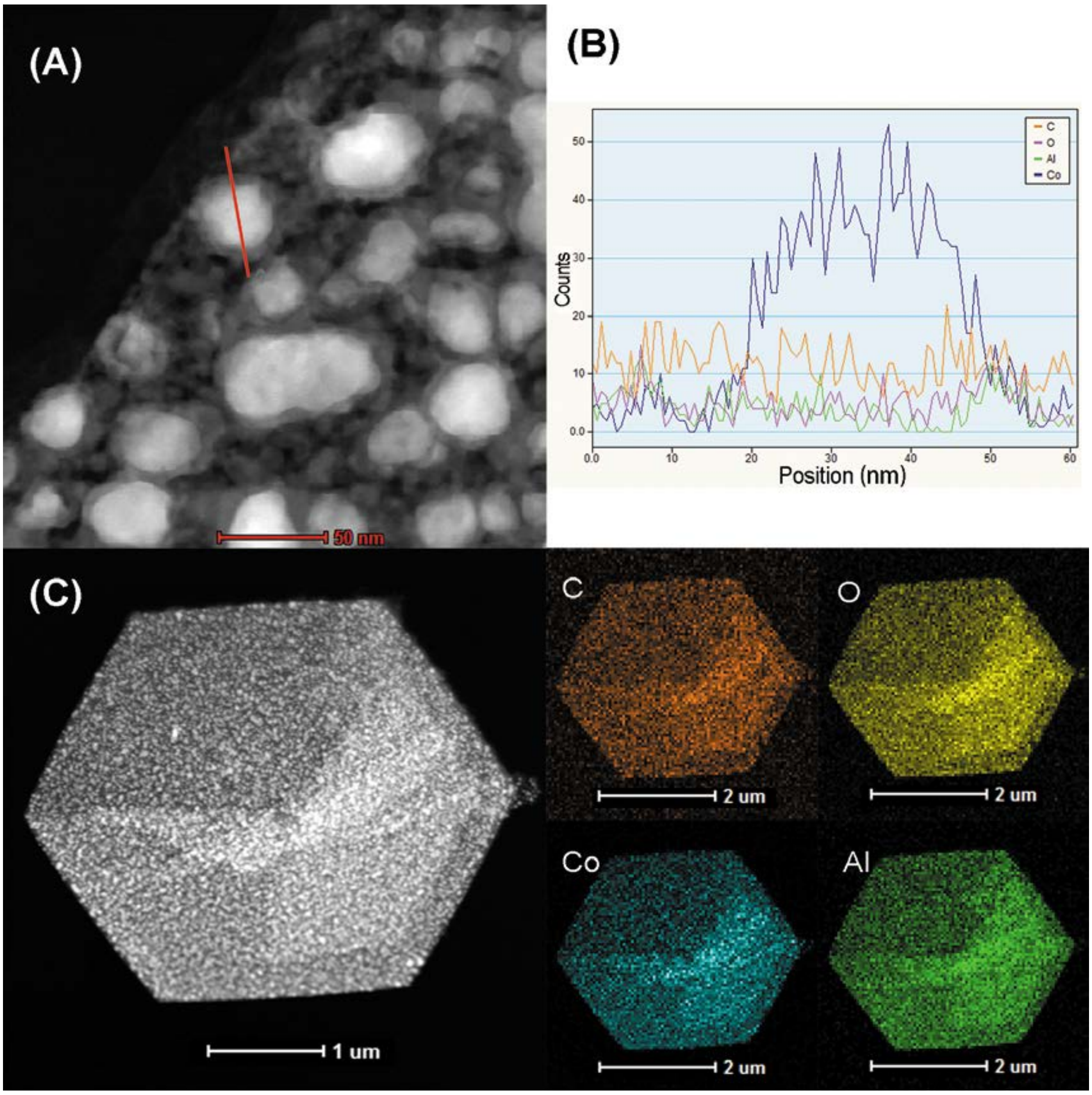

Figure S5: (A) HAADF-STEM image of the NPLs-2.5-800 sample. (B) C, O, Al and Co elemental line profiles along the red line across the sample in (A). (C) Dark-field TEM image of the NPLs-2.5-800 and examination of the corresponding elemental mappings of C, O, Co, and Al.

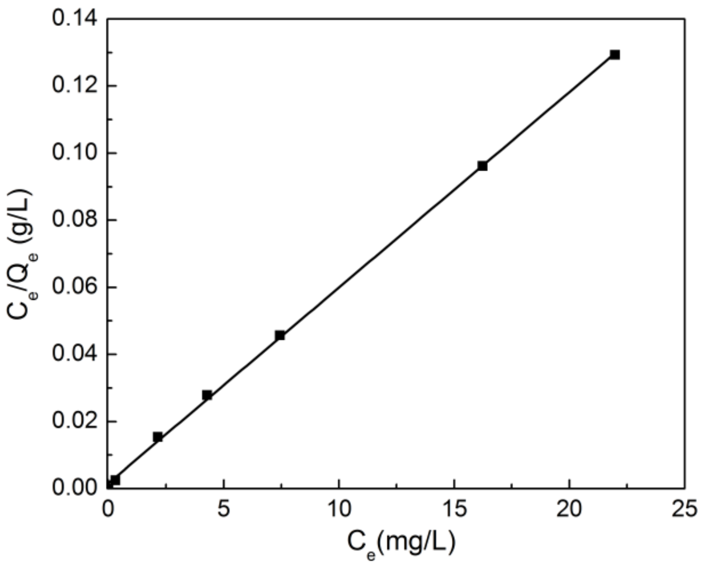

Figure S6: Fitting of the adsorption isotherm of RB on the hexagonal magnetic mesoporous NPLs-2.5-800 by using the Langmuir model.

Table S3: Parameters of adsorption isotherms for removal of RB from water by the hexagonal magnetic mesoporous NPLs-2.5-800.

Isotherms	Parameters		
Langmuir	Q_{max}	K_l	R^2
	172.41	3.05	0.9995

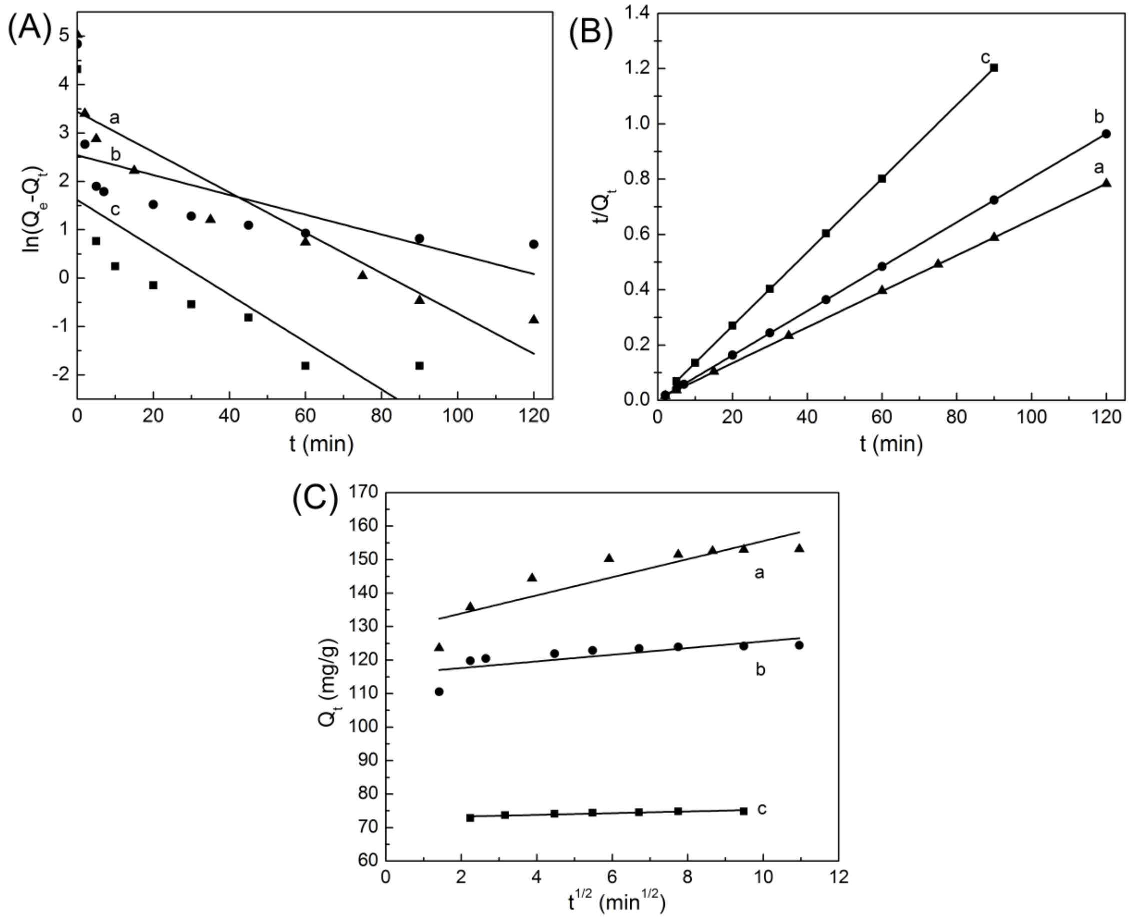

Figure S7: (A) Pseudo-first-order adsorption rates, (B) pseudo-second-order adsorption rates, and (C) intraparticle diffusion rates for RB adsorption onto the hexagonal magnetic mesoporous NPLs-2.5-800 at different initial RB concentrations: (a) 35, (b) 25, and (c) 15 mg/L.

Table S4: The parameters of adsorption kinetic for removal of RB from water by the hexagonal magnetic mesoporous NPLs-2.5-800 at different initial RB concentrations.

Concentration of RB (mg/L)	Pseudo-first-order			Pseudo-second-order			Intraparticle diffusion		
	Q_e	K_1	R^2	Q_e	K_2	R^2	K_3	C	R^2
15	3.19	0.049	0.5982	75.02	0.068	1	0.25	72.78	0.8309
25	12.66	0.021	0.4539	124.69	0.024	1	0.99	115.65	0.5775
35	31.15	0.042	0.8623	154.08	0.008	0.9999	2.70	128.54	0.7861

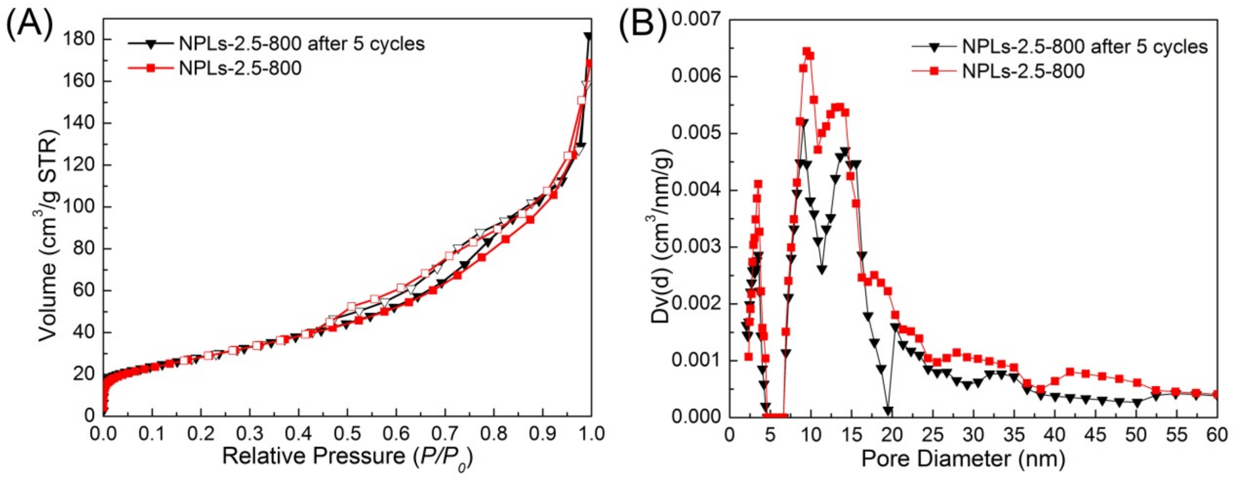

Figure S8: (A) Nitrogen adsorption-desorption isotherms of the NPLs-2.5-800 sample before and after five cycles. (B) Corresponding pore size distributions derived from the adsorption branches using NLDFT method.

Table S5: Comparison of adsorption capacity of various adsorbents for RB dye removal.

Adsorbent	Feedstock	Method used	Q_{max} (mg/g)	Reference
Magnetite/reduced graphene oxide	Fe_3O_4, graphene oxide	Hydrothermal method	4.23	[S1]
Carbon/iron oxide	Activated carbon, iron oxide	Hydrothermal carbonization	47.44	[S2]
C_{16}/SiO_2–Fe_3O_4 nanoparticles	Fe_3O_4, hexadecyl, Si–OH	Solvothermal method	36.50	[S3]
Graphene–Fe_3O_4 nanocomposite	Graphite oxide, PDDA, Fe_3O_4	Chemical co-precipitation	166.40	[S4]
Graphene oxide–Fe_3O_4 nanoparticles	Graphene oxide, $FeCl_3$, $FeCl_2$	Chemically synthesis	44.40	[S5]
Ni@graphene nanocomposites	Graphite oxide, Ni-contained precursor	Ultrasonic process	83.00	[S6]
Oligo–layer graphite nanoflakes	[Bmim][$FeCl_4$], fructose	Ionothermal synthesis	150.00	[S7]
NPLs-2.5-800	CoAl LDH, PDA	Hydrothermal carbonization	172.41	This study

References

[S1] Sun, H.; Cao, L.; Lu, L. *Nano Res*. **2011**, *4*, 550–562.

[S2] Singh, K. P.; Gupta, S.; Singh, A. K.; Sinha, S. *Chem. Eng. J.* **2010**, *165*: 151–160.

[S3] Chang, Y.; Ren, C.; Yang, Q.; Zhang, Z.; Dong, L.; Chen, X.; Xue, D. *Appl. Surf. Sci.* **2011**, *257*, 8610–8616.

[S4] Lu, W.; Wu, Y.; Chen, J.; Yang, Y. *Crystengcomm* **2014,** *16*, 609–615.

[S5] Geng, Z.; Lin, Y.; Yu, X.; Shen, Q.; Ma, L.; Li, Z.; Pan, N.; Wang, X. *J. Mater. Chem.* **2012,** *22*, 3527–3535.

[S6] Zhao, C.; Guo, J.; Yang, Q.; Tong, L.; Zhang, J..; Zhang, J.; Gong, C.; Zhou, J.; Zhang, Z. *Appl. Surf. Sci.* **2015**, *357*, 22–30.

[S7] Xie, Z. L.; Huang, X.; Titirici, M. M.; Taubert, A. *Rsc Adv.* **2014**, *4*, 37423–37430.
